# Une cause rare de déplacement de la sonde de pacemaker: syndrome de Twiddler

**DOI:** 10.11604/pamj.2019.33.107.9051

**Published:** 2019-06-12

**Authors:** Ibn Hadj Amor Hassen, Kammoun Sofiane, Zoghlami Bilel, Zairi Ihsen, Kraiem Sondes

**Affiliations:** 1 Service de Cardiologie, Hôpital Militaire Principal d’Instruction, Tunis, Tunisie; 2 Service de Cardiologie, Hôpital Habib Thameur, Tunis, Tunisie

**Keywords:** Pacemaker, syndrome de Twiddler, sonde de stimulation, Pacemaker, Twiddler’s syndrome, stimulation lead

## Abstract

Le syndrome de Twiddler, une complication rare mais potentiellement létale de l’implantation de stimulateur cardiaque. Il est généralement diagnostiqué dans la première année de l'implantation. Il est caractérisé par un dysfonctionnement de l'appareil en raison de délogement de la sonde de pacemaker suite à l’auto manipulation du patient. Le syndrome de Twiddler a été décrit en 1968 comme une complication de l'implantation du stimulateur. Il a également été rapporté avec les défibrillateurs implantables et les resynchroniseurs cardiaques. Il s'agit du cas d'une vieille dame admise pour syndrome de Twiddler, ce qui a entraîné un dysfonctionnement de stimulateur cardiaque secondaire au déplacement de la sonde du pacemaker. Elle a bénéficié d’un repositionnement de la sonde ainsi que des conseils appropriés.

## Abstract (en)

Twiddler’s syndrome is a rare but potentially lethal complication of pacemaker implantation. It is usually diagnosed within 1 year after implantation. It is characterized by device malfunction caused by pacemaker lead displacement due to twisting of the box on part of the patient. Twiddler’s syndrome has been described by in 1968 as a complication of pacemaker implantation. It has also been reported after defibrillator implantation and cardiac resynchronization therapy. We here report the case of a 89-year old female patient admitted with Twiddler’s syndrome leading to pacemaker malfunction secondary to pacemaker lead displacement. She underwent lead repositioning and received appropriate advice.

## Introduction

Le syndrome de Twiddler ou Twist syndrome correspond à un aspect entortillé ou enroulé de la sonde du pacemaker ou du défibrillateur cardiaque sur elle-même. Ce syndrome, décrit pour la première fois par Bayliss *et al.* en 1968, est le résultat d'une manipulation du dispositif implanté par le patient lui-même, ce qui pourrait conduire au délogement du dispositif. Bien que dans la majorité des cas, le délogement des sondes est indolore, la défaillance du dispositif peut être la cause de graves conséquences.

## Patient et observation

**Figure 1:**
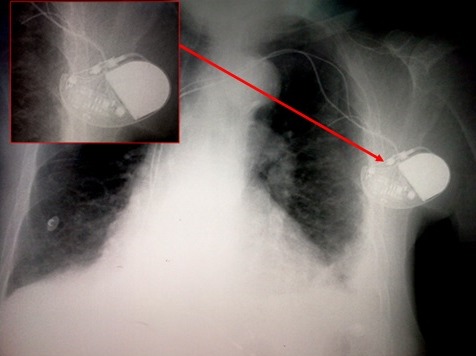
Radiographie du thorax de face montrant l’aspect normal (non enroulé) de la sonde du stimulateur cardiaque après son implantation et avant la survenue du syndrome de Twiddler

**Figure 2:**
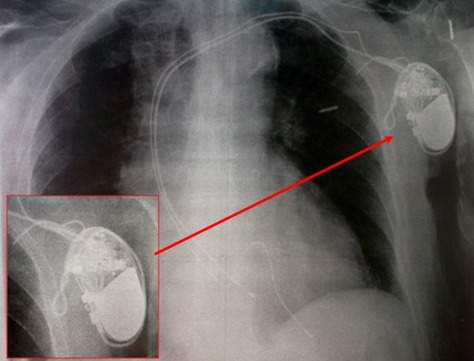
Radiographie du thorax de face montrant le déplacement de la sonde ventriculaire et son enroulement autour du boitier réalisant une spire

**Figure 3:**
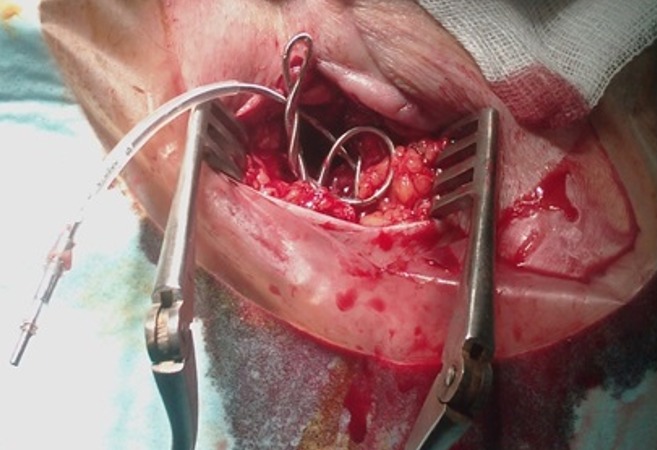
Enroulement de la sonde de stimulation: vue per opératoire avant le repositionnement de la sonde du pacemaker

Une patiente de 89 ans était admise à notre salle d’urgence pour syncope. Son histoire de la maladie commence depuis 1992 par l’implantation d’un stimulateur cardiaque monochambre au niveau de la loge pectorale gauche pour un bloc auriculoventriculaire complet. Ce pacemaker était explanté en 2004, devant l'impossibilité de l'interroger, et a été remplacé par un autre stimulateur double chambre mis au niveau de la loge pectorale controlatérale (droite) avec mise de deux nouvelles sondes ventriculaire et auriculaire. L'ancienne sonde ventriculaire a été gardée en place. Deux mois avant l'épisode actuel, la patiente a été hospitalisée pour infection de la loge de pacemaker. Le matériel infecté a été retiré et un stimulateur cardiaque monochambre a été implanté au niveau de la loge pectorale gauche avec mise d’une nouvelle sonde ventriculaire à fixation passive, procédure qui s'est déroulée sans complication. On note par ailleurs des troubles mnésiques récents. À l’arrivée de la patiente à la salle d'urgence, elle avait un pouls régulier à 36 bpm. L’électrocardiogramme (ECG) a montré un bloc auriculoventriculaire complet avec des spikes ventriculaires ratés. Après stabilisation de l'état de la patient et sa mise sous isoprénaline, la radiographie de thorax a montré un déplacement de la sonde ventriculaire avec un aspect enroulé de la sonde du coté de boitier (Figure 1, Figure 2), cadrant avec un syndrome de Twiddler. L'exploration per-opératoire (Figure 3) documente cet enroulement de la sonde ventriculaire dont l'impédance est restée constante à 400 ohms. La présence d'une sténose du tronc veineux innominé a obligé l'opérateur à garder cette sonde qui a été repositionnée dans l'apex du ventricule droit avec une écoute ventriculaire à 10mV et un seuil ventriculaire à 0,5V. La loge du stimulateur cardiaque a été réduite et le boitier a été attaché au plan musculaire. Les suites post-opératoires ont été simples. La patiente a été adressée chez un neurologue qui a posé le diagnostic d'une démence vasculaire. Après un recul de 6 mois, aucun événement clinique n'a été documenté chez cette patiente.

## Discussion

Le syndrome de Twiddler a été décrit pour la première fois par Bayliss *et al.* en 1968 [1]. Il résulte de la manipulation du dispositif implanté par le patient lui-même et s'intègre habituellement dans le cadre d'une démence ou d'un trouble obsessionnel compulsif [2]. Il se révèle habituellement par l'extériorisation du boitier ou une usure de la sonde. En revanche un déplacement isolé de la sonde de stimulation est moins fréquent. L’altération de la sonde de stimulation associée à son enroulement constitue le « syndrome Twiddler plus ». La fréquence de ce syndrome varie en fonction des séries de 0,7% à 7% [3, 4]. Les facteurs de risque associés au syndrome de Twiddler sont le genre féminin, l'obésité, l'âge et la démence [1, 5], mais il semble que la démence est de loin le facteur de risque le plus important [5], comme c'est le cas de notre observation.

Les patients âgés et obèses semblent être à risque en raison de la présence d'un tissu lâche sous-cutané qui permet le glissement et la rotation du dispositif dans sa poche [6]. La majorité des patients atteints de ce syndrome sont diagnostiqués dans la première année suivant l’implantation. Plusieurs variétés de ce syndrome ont été décrites avec la manipulation d'autres dispositifs comme les défibrillateurs automatiques implantables [7] et les chambres implantables [8]. Afin de prévenir ce syndrome, il est recommandé de fixer le dispositif implanté au plan musculaire [9], la réduction de la taille de la loge et dans certains cas par la confection d'une poche en dacron autour du dispositif implanté [10]. L'utilisation d'une sonde à fixation active est recommandée par certaines équipes [11]. La récidive de ce syndrome est rare [10] et c’est souvent l’apanage des patients ayant une symptomatologie psychiatrique avérée, mal ou non traitée. Notre observation présente la particularité de la survenue de syndrome de Twiddler après une longue histoire de pacing. Ce syndrome est révélateur d'une démence jusque-là non diagnostiquée.

## Conclusion

Le syndrome de Twiddler est une cause rare de déplacement de sonde de stimulation. Son intrication avec des antécédents ou des maladies psychiatriques incite le praticien à procéder à une évaluation psychiatrique du malade présentant ce syndrome. La fixation du dispositif au plan musculaire est une mesure simple qui prévient dans la plupart des cas la récidive de ce syndrome.

## Conflits d’intérêts

Les auteurs ne déclarent aucun conflit d’intérêts.
